# Supplementary material for: Measuring Zak phase in room-temperature atoms
Source: Light Sci Appl. 2022 Oct 9;11:291. doi: 10.1038/s41377-022-00990-7 (PMC9548506; doi:10.1038/s41377-022-00990-7)
Supplement: Supplementary file 1 — Supplementary Notes: Measuring Zak phase in room-temperature atoms [file 41377_2022_990_MOESM1_ESM.pdf]

Supplementary Notes: Measuring Zak phase in room-temperature atoms

Ruosong Mao[1,5], Xingqi Xu[1,5], Jiefei Wang[1,5], Chenran Xu[1],
Gewei Qian[1], Han Cai[2,1,*], Shi-Yao Zhu[1,3], and Da-Wei Wang[1,3,4,†]
[1] *Interdisciplinary Center for Quantum Information,
State Key Laboratory of Modern Optical Instrumentation,
and Zhejiang Province Key Laboratory of Quantum Technology and Device,
Department of Physics, Zhejiang University, Hangzhou 310027, China*
[2] *College of Optical Science and Engineering,
Zhejiang University, Hangzhou, 310027, China*
[3] *Hefei National Laboratory, Hefei 230088, China*
[4] *CAS Center for Excellence in Topological Quantum Computation,
University of Chinese Academy of Sciences, Bejing 100190, China*
[5] *These authors contributed equally: Ruosong Mao,
Xingqi Xu, and Jiefei Wang.*
[*] *e-mail: hancai@zju.edu.cn.*
[†] *e-mail: dwwang@zju.edu.cn.*
(Dated: September 21, 2022)

CONTENTS

1. Experimental setup 2

2. The derivation Eq. (5) and its validation 3

3. The SSH model, the Zak phase, and its gauge dependence 5

4. Absorption coefficient at different temperatures 6

5. Band Centers and anti-crossings in the absorption spectra 7

6. How to distinguish the spectroscopic features? 8

7. Supplementary figures 10

References 10

1. EXPERIMENTAL SETUP

The full experimental setup is shown in Fig. S1. Lasers from a Ti:Sapphire laser (Spectra-Physics, Matisse C) and a diode laser (Toptica, DLC TA Pro 795) are tuned to frequencies near the ^{87}Rb D1 line (795nm). Two single-mode fibers are used to prepare Gaussian beams. The setup includes three modules for the saturated absorption spectroscopy (SAS), the EIT and the superradiance lattice (SL), as shown in Fig. S2. In the experiment, SAS and EIT are used to obtain the frequencies of the optical fields and the absorption spectra of the SL are used to reconstruct the Zak phases.

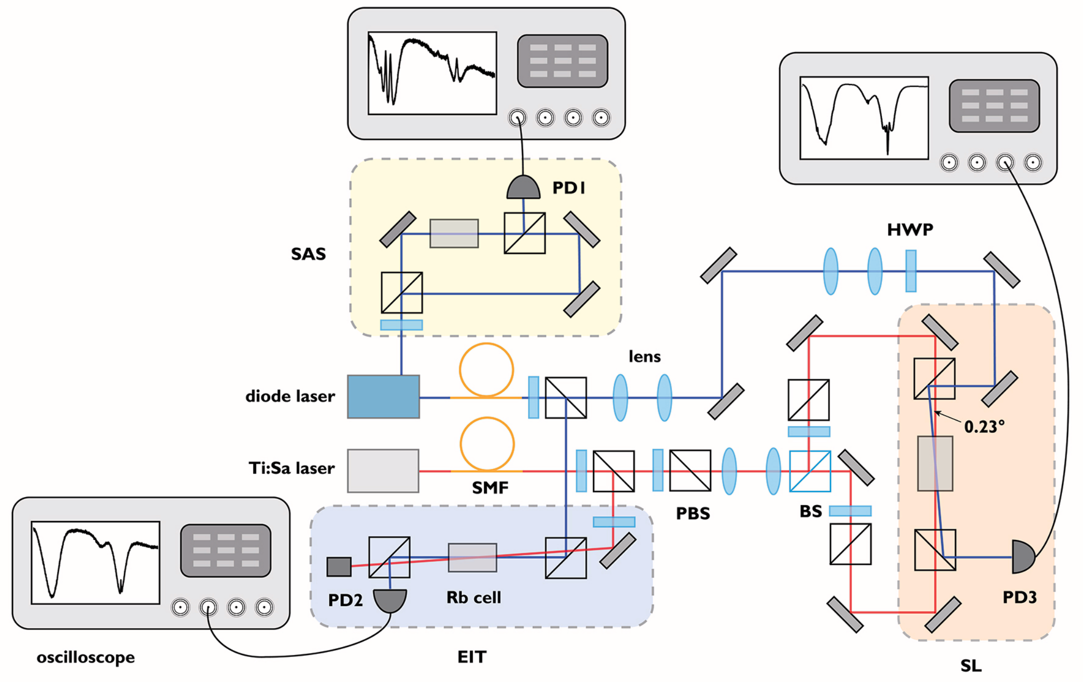

Figure S1. Experimental setup. The yellow, blue and orange zones are the optical modules for SAS, EIT and SL, correspondingly. PBS: polarization beam splitter; BS: 50/50 beam splitter; SMF: single-mode fiber; HWP: half-wave plate; PD: photodiode detector.

We use the SAS (the blue line in Fig. S2) to calibrate the resonant frequency of the atomic transitions with inhomogeneous broadening. A strong pump beam (30 mW) passes through a rubidium cell of natural abundance and saturates the population in the excited state. A weak probe beam (30 μW) propagates in the opposite direction, with its transmission spectrum being detected by a photodiode (PD1), and recorded on an oscilloscope.

We use a Λ-type EIT spectrum (red line in Fig. S2) to detect and monitor the frequency of the coupling field. A strong pump field (60 mW) from the Ti:Sapphire laser couples the atomic transition $|g\rangle \equiv |5^2S_{1/2}, F = 1\rangle$ to $|b\rangle \equiv |5^2P_{1/2}, F = 2\rangle$, and a co-propagating weak field (22 μW) from a diode laser is used to probe the transition from $|b\rangle$ to $|a\rangle \equiv |5^2S_{1/2}, F = 2\rangle$. There is a small angle between the probe and coupling fields. The transmission of the weak probe field is collected by a photodiode (PD2). A transparent point appears when the two-photon resonance condition is satisfied, i.e., $\nu_c - \omega_{ba} = \nu_p - \omega_{bg}$. Combining the data from the SAS and EIT modules, we obtain the detuning of the coupling field Δ_c.

We use a standing-wave-coupled EIT configuration to construct one-dimensional SLs (the black line in Fig. S2). A 2 cm long vapor cell is filled with natural abundance rubidium atoms. We heat the vapor cell to 70 °C to increase the density of atoms to obtain optimized absorption spectra with enhanced features in absorption dips and peaks. Two identical beams (coupling 1 and 2) are prepared as follows. The Ti:Sapphire laser is stabilized by the Pound-Drever-Hall technique, ensuring the stability of the laser power and frequency during the one-time data collection. The laser beam from the optical cavity is coupled into a single-mode fiber for spatial mode filtering to obtain a Gaussian beam. A telescope system is used to focus the beam diameter to 1 mm. The beam is then split by a 50/50 BS to obtain the two plane wave components of the standing wave coupling field. Since the beams slightly diverge in propagation, the

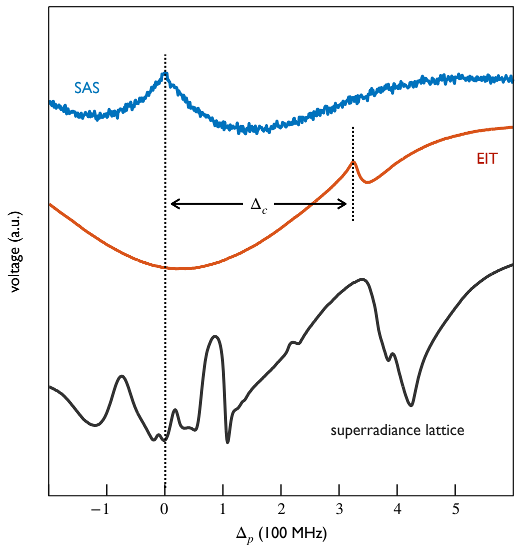

Figure S2. Spectra of saturated absorption, electromagnetically induced transparency and superradiance lattice. Blue line: SAS of a vapor cell of natural abundance rubidium. The peak indicates the atomic transition $|5^2S_{1/2}, F=1\rangle \rightarrow |5^2P_{1/2}, F=2\rangle$ of the ^{87}Rb D1 line. Red line: An EIT spectrum used to determine the frequency of the coupling field, where the transparency peak indicates the coupling field detuning Δ_c. Black line: A typical transmission spectrum of a superradiance lattice.

light paths of two beams are carefully controlled to guarantee that the two standing wave components have the same beam size in the Rb cell.

A fiber-coupled diode laser provides the probe beam. The beam size is controlled by a telescope system, in which two groups of lenses with 50/100 and 100/50 mm focal lengths are used to expand and reduce the beam size, respectively. With this telescope system we obtain a 0.5mm diameter probe beam to ensure that the beam spatially overlaps with the coupling fields within the vapor cell. A High-gain PD3 (Thorlabs, PDA36A2) is used to record the probe field absorption spectra through the SL. The probe beam has a power 12 μW and a small incident angle 0.23° with respect to the axis of the standing waves. The polarizations of the coupling and the probe beams are perpendicular to each other.

2. THE DERIVATION EQ. (5) AND ITS VALIDATION

Here we derive the energies of the WSLs. The Hamiltonian of a two-level atom coupled with a standing wave in the interaction picture is,

$$V_s = \Delta_c |a\rangle\langle a| + [(\Omega_1 e^{ik_c x} + \Omega_2 e^{-ik_c x})|a\rangle\langle b| + H.c.],$$

which can be considered as the Bloch Hamiltonian of the tight-binding Hamiltonian H_s. The eigenenergies $\lambda_\pm$ and eigenstates $u_\pm$ of V_s are [4],

$$\begin{aligned} \lambda_\pm &= \frac{\Delta}{2} \pm \sqrt{\left(\frac{\Delta}{2}\right)^2 + \Omega_1^2 + \Omega_2^2 + 2\Omega_1\Omega_2\cos(2k_c x)}, \\ u_+ &= (u_{+,a},\ u_{+,b}) = \left(\cos(\frac{\eta}{2}),\ e^{-i\phi}\sin(\frac{\eta}{2})\right), \\ u_- &= (u_{-,a},\ u_{-,b}) = \left(-\sin(\frac{\eta}{2}),\ e^{-i\phi}\cos(\frac{\eta}{2})\right), \end{aligned}$$

where

$$\tan(\eta) = \frac{2|\Omega_1 e^{ik_c x} + \Omega_2 e^{-ik_c x}|}{\Delta}, \quad e^{i\phi} = \frac{\Omega_1 e^{ik_c x} + \Omega_2 e^{-ik_c x}}{|\Omega_1 e^{ik_c x} + \Omega_2 e^{-ik_c x}|}.$$

We introduce the creation operator

$$d^\dagger_{\pm,x} = \frac{1}{\sqrt{N}} \sum_j \left(u_{\pm,a} e^{ik_{2j}x} a^\dagger_{2j} + u_{\pm,b} e^{ik_{2j+1}x} b^\dagger_{2j}, \right). \tag{S1}$$

The linear potential term H_f induces Bloch oscillation of a general excitation,

$$\Psi^\dagger = C_1 d^\dagger_{+,x'} + C_2 d^\dagger_{-,x'},$$

which moves in the Brillouin zone $x' = x + vt$. Here C_1 (C_2) is the probability amplitude of the excitation in the upper (lower) band. The dynamics of the excitation can be solved according to the Heisenberg equation $d\Psi^\dagger/dt = i[H_s + H_f, \Psi^\dagger]$. Neglecting the interband (Landau-Zener) transitions, we only need to analyze the excitation dynamics in the same band [5]. Here we only focus on the component in the upper band,

$$\frac{d}{dt} C_1 d^\dagger_{+,x'} = i\left[H_s + H_f, C_1 d^\dagger_{+,x'}\right].$$

The left-hand side reads $\frac{dC_1}{dt} d^\dagger_{+,x'} + C_1 \frac{d}{dt} d^\dagger_{+,x'}$, where

$$\begin{aligned} \frac{d}{dt} d^\dagger_{+,x'} &= \frac{1}{\sqrt{N}} \sum_j \left[\left(ik_{2j}v + \frac{\partial u_{+,a}}{\partial t} \right) e^{ik_{2j}(x+vt)} a^\dagger_{2j} + \left(ik_{2j+1}v + \frac{\partial u_{+,b}}{\partial t} \right) e^{ik_{2j+1}(x+vt)} b^\dagger_{2j+1} \right] \\ &= \frac{v}{\sqrt{N}} \sum_j \left[\left(ik_{2j} + \frac{\partial u_{+,a}}{\partial x'} \right) e^{ik_{2j}x'} a^\dagger_{2j} + \left(ik_{2j+1} + \frac{\partial u_{+,b}}{\partial x'} \right) e^{ik_{2j+1}x'} b^\dagger_{2j+1} \right]. \end{aligned}$$

and right-hand side reads

$$\left[H_s + H_f,\ d^\dagger_{+,x'}\right] = \frac{\delta}{\sqrt{N}} \sum_j \left[2j u_{+,a} e^{ik_{2j}x'} a^\dagger_{2j} + (2j+1) u_{+,b} e^{ik_{2j+1}x'} b^\dagger_{2j+1} \right] + \lambda_+ d^\dagger_{+,x'}.$$

By comparing the two sides we obtain the dynamic equation of the probability amplitude C_1,

$$-i\frac{dC_1}{dt} = C_1 \left(\lambda_+ + iv u^\dagger_{+,a} \partial_{x'} u_{+,a} + iv u^\dagger_{+,b} \partial_{x'} u_{+,b} \right).$$

The nth WSL energy can be obtained by the phase accumulated after the particle moves across a whole Brillouin zone,

$$\begin{aligned} E^{[n]}_+ &= \frac{k_c}{\pi} \int_0^{\pi/k_c} dx' \left(\lambda_+ + iv u^\dagger_{+,a} \partial_{x'} u_{+,a} + iv u^\dagger_{+,b} \partial_{x'} u_{+,b} \right) \\ &= \epsilon_+ + \frac{\delta}{\pi}(\theta_+ + 2n\pi), \end{aligned}$$

in which $\delta = k_c v$, $\epsilon_+ = \frac{k_c}{\pi} \int_0^{\pi/k_c} dx \lambda_+$ is the energy of the band center, and the Zak phase of the upper band is $\theta_+ = i\int_0^{\pi/k_c} dx u^\dagger_+ \partial_x u_+$ modulo 2π.

Eq. (5) holds in the weak force approximation, which can break down in two different ways.

- **Resonant coupling between Wannier functions from different bands.**
  Eq. (5) is not valid near the anti-crossings. The avoided regime is determined by the coupling strength between the Wannier functions, which depends on the overlapping of the wavefunctions. Since the Wannier functions are localized, the overlap and coupling strength are generally determined by the distance between them and the band width. As shown in Fig. S3, the avoided regime gets wider when the band width increases from (a) to (d). In particular, in Fig. S3(d), the coupling between the next-nearest-neighboring unit cells is large enough to induce visible anti-crossings.

- **The competition between the lattice Hamiltonian H_s and the linear potential H_f**
  Eq. (5) is obtained in the limit when H_f is perturbative. A rough estimation is that the force δ should be much less than the difference of the two band centers $\epsilon_+ - \epsilon_-$. In the opposite limit, the eigenenergy is dominated by H_f, which exhibits an integer slope from the definition. To clarify this point, we notice that the slope of

the eigenstates is deviated from $\partial E_{\pm}^{[0]}/\partial\delta = 0.5$ (the dashed lines) when $|\delta|$ is comparable to $\Omega_1 = \epsilon_+ = -\epsilon_-$ (Fig. S3(a)) without anti-crossings. The slope deviation can be analytically obtained in the dimerization limit of the SSH model, $\Omega_2 = \Delta_c = 0$,

$$E_{\pm}^{[0]}(\delta) = \frac{\delta}{2} \pm \sqrt{\frac{\delta^2}{4} + \Omega_1^2},$$
$$\frac{\partial E_{\pm}^{[0]}}{\partial \delta} = \frac{1}{2} \pm \frac{\delta/4}{\sqrt{\delta^2/4 + \Omega_1^2}}.$$

As we expect, the local slope $\partial E_{\pm}^{[0]}/\partial\delta = 0.5$ in the limit of $|\delta| \ll \Omega_1$ and

$$\frac{\partial E_{+}^{[0]}}{\partial \delta}(\delta \gg \Omega_1) = 1,$$
$$\frac{\partial E_{-}^{[0]}}{\partial \delta}(\delta \gg \Omega_1) = 0.$$

The linear-dependence deviation of the lower band in Fig. S3(a) can be valued by two quantities in Fig. S3(e): the local slope $\partial E_{-}^{n}/\partial\delta$ and the overall slope of a line connecting $E_{-}^{n}(\delta)$ and the lower band center. Since we presume a linear dependence in our Zak phase reconstruction (Eq. 7 in the main text), the error of the overall slope is more relevant in our method. We notice that the overall slope is approximately 0.4 when $\delta \approx \Omega_1$, which is lower than the theoretical predicted value 0.5.

In conclusion, Eq. (5) holds valid when H_f can be treated as a perturbation without anti-crossings. However, the anti-crossing points characterized by dips are approximately on the lines determined by Eq. (5), which helps us to extract the slopes.

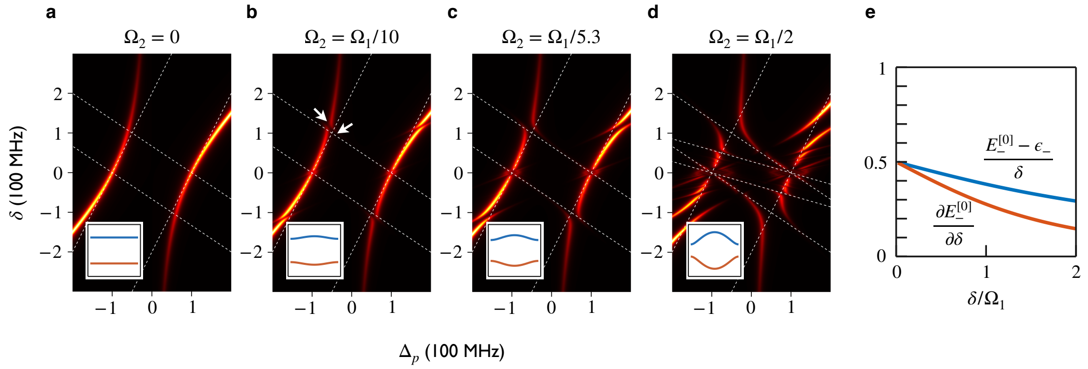

Figure S3. (a-d) The energy-force diagram of WSL with different Ω_2/Ω_1 and fixed band centers $\epsilon_\pm = \pm 100$ MHz. Insets: band structure. (e) The local derivative $\partial E_{-}^{n}/\partial\delta$ and the overall slope of a line connecting $E_{-}^{n}(\delta)$ and the lower band center of (a).

3. THE SSH MODEL, THE ZAK PHASE, AND ITS GAUGE DEPENDENCE

The discovery of SSH model originates from the study of the exotic transport property of the polymer polyacetylene. Here we numerically compute the energy spectra of the two topologically distinct configurations of polyacetylene molecules in Fig. S4. They exhibit the same band structure but are distinguished by the topological edge states. The model can be further simplified by only taking the valence and conduction bands (highlighted in gray) into consideration. In their seminal work [1], Su, Schrieffer, and Heeger proposed the one-dimensional tight-binding model with alternative nearest-neighbor hopping strengths, which is one of the most important models of topological physics.

The Zak phase is a gauge-dependent quantity, depending on the choice of the unit cell. We consider a translation

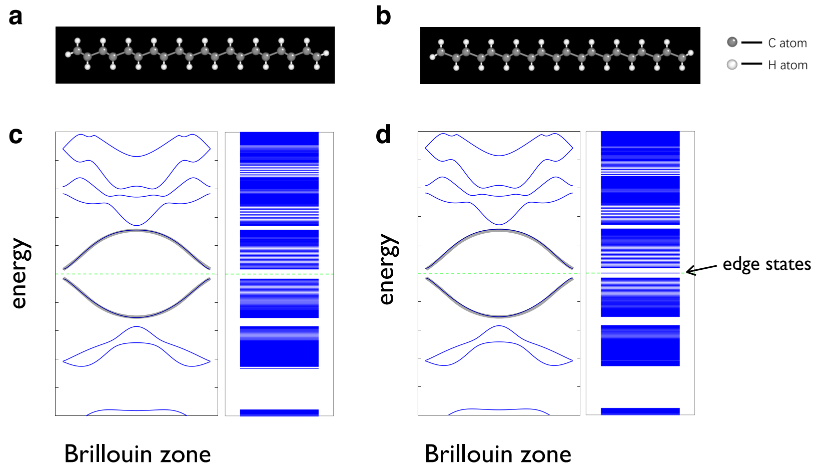

Figure S4. (a,b) The chemical structures of the two configurations of polyacetylene molecules. (c,d) The two configurations show the same band structure in periodic boundary condition (left panels). Only one configuration is featured with topological edge states in open boundary condition with 51 unit cells (right panels). The lengths of the carbon-carbon bonds are set as 1.756 Å and 1.633 Å, respectively. The dashed lines indicate the zero energy point in the spectra. The valence and conduction bands are highlighted in gray. Packages Nanoskim [2] and Nanodcal [3] are used in the calculation.

in momentum space $k \to k + \kappa$ to the creation operator (Eq. S1),

$$d^\dagger_{+,x} = \frac{1}{\sqrt{N}} \sum_j (u_{+,a} e^{i2jk_c x} a^\dagger_{2j} + u_{+,b} e^{i(2j+1)k_c x} b^\dagger_{2j+1}$$
$$\to \frac{1}{\sqrt{N}} \sum_j (\tilde{u}_{+,a} e^{i(2jk_c - \kappa)x} a^\dagger_{2j} + \tilde{u}_{+,b} e^{i((2j+1)k_c - \kappa)x} b^\dagger_{2j+1}).$$

After such a physically trivial translation (which is a gauge transformation), the new eigenstates $\tilde{u}_+ = (\tilde{u}_{+,a}, \tilde{u}_{-,b})$ are related to the old ones by

$$\tilde{u}_+ = u_+ e^{i\kappa x}.$$

The Zak phase is calculated in the new basis

$$\tilde{\theta}_+ = i \int_0^{\pi/k_c} dx \tilde{u}^\dagger_+ \partial_x \tilde{u}_+ = i \int_0^{\pi/k_c} dx (u^\dagger_+ \partial_x u_+ + i\kappa) = \theta_+ - \pi\kappa/k_c.$$

Similarly, we have $\tilde{\theta}_- = \theta_- - \pi\kappa/k_c$. It indicates that the value of Zak phase depends on the chosen gauge while the difference between the two bands is gauge invariant $\tilde{\theta}_+ - \tilde{\theta}_- = \theta_+ - \theta_-$.

Here we must emphasize a difference between the SL and the conventional lattice when we consider the zero energy reference of lattice potential. In conventional lattices we can set arbitrary energy as the zero energy reference without changing any physical observable, since only the relative value matters for potential energy. However, in the SL, the zero-energy point of the linear potential H_f due to the Doppler shift is an observable, which has a definite physical meaning and can be measured. In other words, we do not have a gauge degree of freedom and Zak phases are observables in our experiment. The SLs investigated here correspond to conventional lattices with a definite gauge.

4. ABSORPTION COEFFICIENT AT DIFFERENT TEMPERATURES

In order to illustrate the influence of temperature, we show the experimental transmission spectra, which are used to obtain the absorption coefficient, of the Semenoff insulator in Fig. S5(a). Apart from the overall amplitude difference, we notice that the peaks and dips have the same positions for different temperatures. It shows that our scheme does not require a special temperature as long as the velocity distribution is large enough to cover all the relevant anti-crossings.

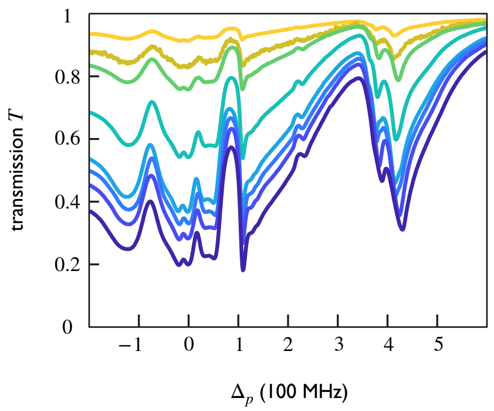

Figure S5. The transmission spectra of the Semenoff insulator with $\Delta_c = 304$MHz for different temperatures, 26.7°C, 35.1°C, 40.1°C, 47.6°C, 55.6°C, 58.1°C, 60.2°C, 62.4°C, from up to down. Other parameters are the same as in Fig. 3(a) in the main text.

5. BAND CENTERS AND ANTI-CROSSINGS IN THE ABSORPTION SPECTRA

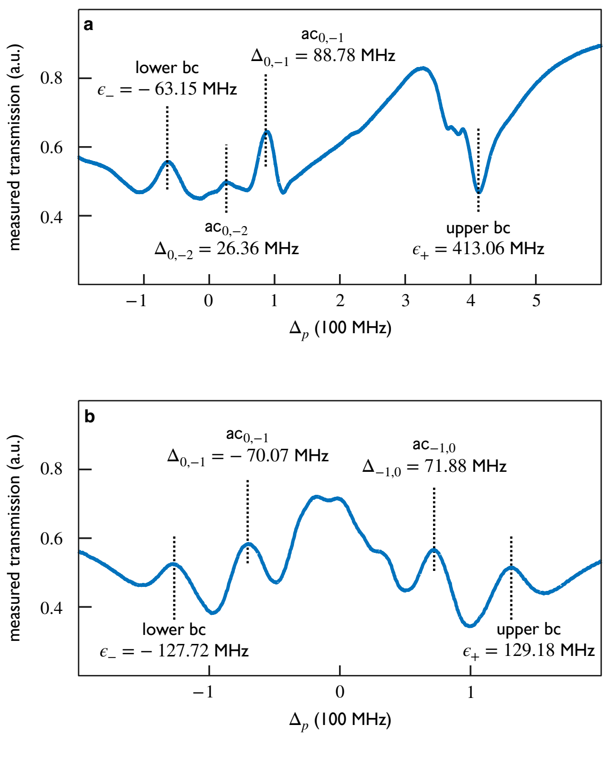

Figure S6. Transmission spectra for **(a)** the Semenoff insulator with $\Delta_c = 304$MHz and **(b)** the SSH model with $\Omega_1/\Omega_2 = 3.23$. Other parameters are the same as in Fig. 3 (a) and (b) in the main text.

We show how to extract the positions of band centers $\epsilon_\pm$ and anti-crossings $\Delta_{n,m}$ from the absorption spectra in two typical cases: the Semenoff insulator in Fig. S6(a) and the SSH model in Fig. S6(b). Based on the agreement between the numerical simulation and experimental data of the absorption spectra (e.g., in Fig. 2(d)-(f)), we attribute each peak and dip to the corresponding anti-crossing and band center. We measure the local extrema (marked by dash lines in Fig. S6) as the values of $\Delta_{n,m}$ and $\epsilon_\pm$. We list the data of four independent runs of experiment in Table. I.

Semenoff insulator								
	ϵ_- (MHz)	$\Delta_{0,-2}$ (MHz)	$\Delta_{0,-1}$ (MHz)	ϵ_+ (MHz)	$R_{0,-2}$	$R_{0,-1}$	θ_+ (π)	θ_- (π)
Dataset 1	-63.15	26.36	88.78	413.06	0.188	0.319	0.045	0.916
Dataset 2	-64.48	26.47	88.18	411.02	0.191	0.321	0.005	0.943
Dataset 3	-63.77	25.27	87.95	416.19	0.186	0.316	0.042	0.904
Dataset 4	-64.48	25.33	89.74	410.80	0.189	0.324	0.107	0.907
Average value	-63.97	25.86	88.66	412.77	0.188	0.320	0.050	0.918
SSH model								
	ϵ_- (MHz)	$\Delta_{0,-1}$ (MHz)	$\Delta_{-1,0}$ (MHz)	ϵ_+ (MHz)	$R_{0,-1}$	$R_{-1,0}$	θ_+ (π)	θ_- (π)
Dataset 1	-127.72	-70.07	71.88	129.18	0.224	0.777	0.445	0.449
Dataset 2	-131.71	-75.27	67.01	126.11	0.219	0.771	0.466	0.430
Dataset 3	-125.30	-70.56	73.96	128.46	0.216	0.785	0.429	0.433
Dataset 4	-125.41	-69.33	73.59	128.57	0.221	0.784	0.428	0.446
Average value	-127.54	-71.31	71.61	128.08	0.220	0.780	0.442	0.440

Table I. Data of four runs of experiment with identical parameters in Fig. S6.

6. HOW TO DISTINGUISH THE SPECTROSCOPIC FEATURES?

After analysing the spectra of Hamiltonians with various parameters, we conclude the following features that can assist our assimilation of the dips and peaks. The SLs in the current study have two bands and their absorption spectra are generally featured with four dips, of which two are associated to the band centers and the other two major anti-crossings. Only in a special case with zero Zak phase, a band center is featured by a peak. To distinguish these spectroscopic features, we need to understand their physical origin and a rough estimation of the Zak phases.

- **Band centers → dips in the far left and far right**
  The band centers are generally characterized by dips in the far left and far right. The anti-crossings beyond the band centers have no signatures on the absorption spectra. The dips induced by the band centers are owing to the Stark localization effect. In Fig S7, we zoom in the energy-force spectrum near the band center and zero force, e.g., in Fig. 2c of the revised manuscript. The spectrum can be separated in two regimes. When $|\delta| \lesssim E_w$ with E_w being the band width, the SL is described by extended Bloch states, with a PDOS similar to that of a 1D tight-binding lattice, i.e., lower in the middle and higher on the two sides. When $|\delta| \gtrsim E_w$, the PDOS is dominated by Wannier-Stark ladders (WSLs), with concentrated PDOS larger than those of Bloch states. Thus a spectrum dip is formed at the band center.

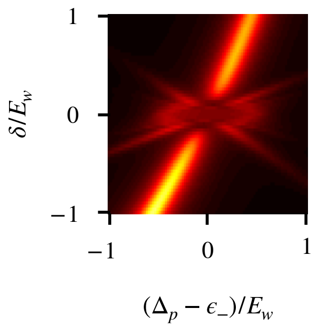

Figure S7. The energy-force diagram near the band center and zero force. The extended Bloch states when $|\delta| \lesssim E_w$ leads to an extended distribution of the PDOS, which gives a dip in absorption spectrum.

- **Anti-crossings → dips between the band centers**
  Between the two band centers, the anti-crossings of WSLs lead to dips in the spectrum because states are not available in the energy gap. Since we measure the PDOS of the state $|\tilde{b}_1\rangle$ in the 0th unit cell, the featured anti-crossings are associated to the Wannier functions localized in 0th and the neighboring −1th unit cells, i.e., $\Delta_{-1,0}$ and $\Delta_{0,-1}$.

- **How to distinguish different anti-crossings?**
  The dips can be related to the anti-crossings with a rough estimation of the Zak phases, i.e., $0 < \theta_\pm < \pi$ or

$\pi < \theta_\pm < 2\pi$, depending on the sign of $\Omega_1 - \Omega_2$ in the Rice-Mele model. As shown in Fig. S8, it helps us to locate the Wannier functions in the left or right half of the unit cells, which determines the value of the slopes and associates the dips to corresponding anti-crossings from a geometric viewpoint.

For convenience, we have the following table to identify dips from left to right in the spectra.

<table>
<tr><td colspan="4">1st dip $\rightarrow$ lower band center ϵ_-</td></tr>
<tr><td colspan="4">4th dip $\rightarrow$ upper band center ϵ_+</td></tr>
<tr><td rowspan="2">2nd dip $\rightarrow$</td><td>anti-crossing $\Delta_{0,-1}$</td><td rowspan="2">when</td><td>$0 < \theta_\pm < \pi$</td></tr>
<tr><td>anti-crossing $\Delta_{-1,0}$</td><td>$\pi < \theta_\pm < 2\pi$</td></tr>
<tr><td rowspan="2">3rd dip $\rightarrow$</td><td>anti-crossing $\Delta_{-1,0}$</td><td rowspan="2">when</td><td>$0 < \theta_\pm < \pi$</td></tr>
<tr><td>anti-crossing $\Delta_{0,-1}$</td><td>$\pi < \theta_\pm < 2\pi$</td></tr>
</table>

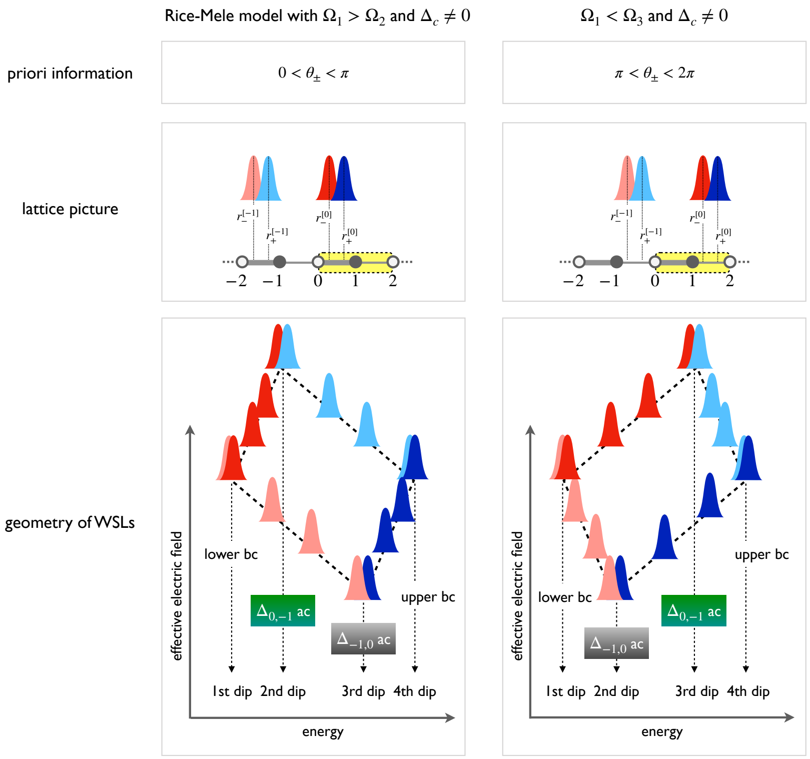

Figure S8. Among the four dips, the two dips associated to band centers are located on the left and right sides while the ones associated to anti-crossings are in the middle. To distinguish $\Delta_{-1,0}$ and $\Delta_{0,-1}$, a rough estimation of the Zak phases is necessary. bc: band centers. ac: anti-crossings. The lower (upper) band Wannier functions are colored in red (blue).

- **Special case: with zero Zak phase the right band center is charaterized by a peak in the spectrum** We plot several spectra with diffrent Zak phases in Fig. S9 to explain that one of the band centers in Fig. 2a is characterized by a peak in the spectrum. When we decreasing θ_+, the WSL $E_+^{[0]}(\delta) = \epsilon_+ + \theta_+\delta/\pi$ turns upright in the energy-force diagram, making the dips of the band center ϵ_+ and the anti-crossing $\Delta_{-1,0}$ get closer. Especially, when $\theta_+ = 0$, the dips merge with an upright WSL, resulting in a peak due to the huge DOS.

  The correspondence between zero Zak phase and spectroscopic peaks can be further clarified by revisiting the zero energy reference of lattice potential in Sec. 3. We add a reference energy V_0 to H_f to obtain a new potential energy (see Fig. S10(a)),

$$H_f' = H_f + V_0,$$

  with

$$V_0 = -\frac{\delta}{2}\sum_j [a_{2j}^\dagger a_{2j} + b_{2j+1}^\dagger b_{2j+1}].$$

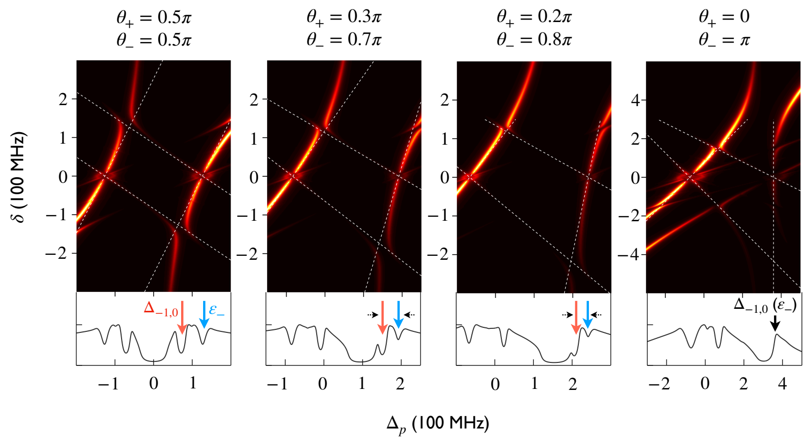

Figure S9. From left to right, the Zak phase of the upper band θ_+ decreases. The dips of band center ϵ_+ and the anti-crossing $\Delta_{-1,0}$ merge when the Zak phase is zero, leading to a peak in the spectrum.

In conventional lattices V_0 is a gauge and can be introduced by trivially redefining the zero potential energy. Conventional lattices with H_f and H'_f have the same physical observables. However, SLs with potential energies H_f and H'_f have different energy spectra. In Fig. S10 (b) and (c), we plot the energy-force diagrams of the SSH model and the Semenoff insulator with both gauges, which have different energy spectra with Zak phases being related by

$$\theta'_\pm = \theta_\pm - 0.5\pi.$$

In particular, it is interesting to note that with H'_f, the SSH model have band centers characterized by peaks in the spectrum due to the vertical WSLs with zero Zak phases. Therefore, whenever we have a zero Zak phase, we obtain a vertical WSL which has a huge DOS located on the band center in the spectrum.

7. SUPPLEMENTARY FIGURES

Here we present the WSL in the energy-force ($\Delta_p - \delta$) diagram of the data in Fig. 3. As shown in Fig. S11(a), when we decrease Δ_c, the energy gap of the anti-crossing widens and higher-order anti-crossings appear, e.g., $\mathrm{ac}_{1,-1}$ in the line with $\Delta_c = 174$ MHz. It is also discernible in the WSL diagrams in Fig. S11(b). For the SSH model in Fig. S12, the coupling between WSLs from different band increases when we change the ratio between the two coupling fields from nearly dimerization ($\Omega_1/\Omega_2 \gg 1$) to topological phase transition ($\Omega_1 \approx \Omega_2$).

[1] W. P. Su, J. R. Schrieffer, and A. J. Heeger, Solitons in polyacetylene, Phys. Rev. Lett. **42**, 1698 (1979).

[2] M. Harb et al., Quantum transport modelling of silicon nanobeams using heterogene-ous computing scheme, J. Appl. Phys. **119**, 124304 (2016).

[3] J. Taylor, H. Guo, and J. Wang, Ab initio modeling of quantum transport properties of molecular electronic devices Phys. Rev. B **63**, 245407 (2001).

[4] M. Yahyavi and B. Hetényi, Reconstruction of the polarization distribution of the Rice-Mele model, Phys. Rev. A. **95**, 062104 (2017).

[5] M. Atala, M. Aidelsburger, J. T. Barreiro, D. Abanin, T. Kitagawa, E. Demler, and I. Bloch, Direct measurement of the Zak phase in topological Bloch bands, Nat. Phys. **9**, 795 (2013).

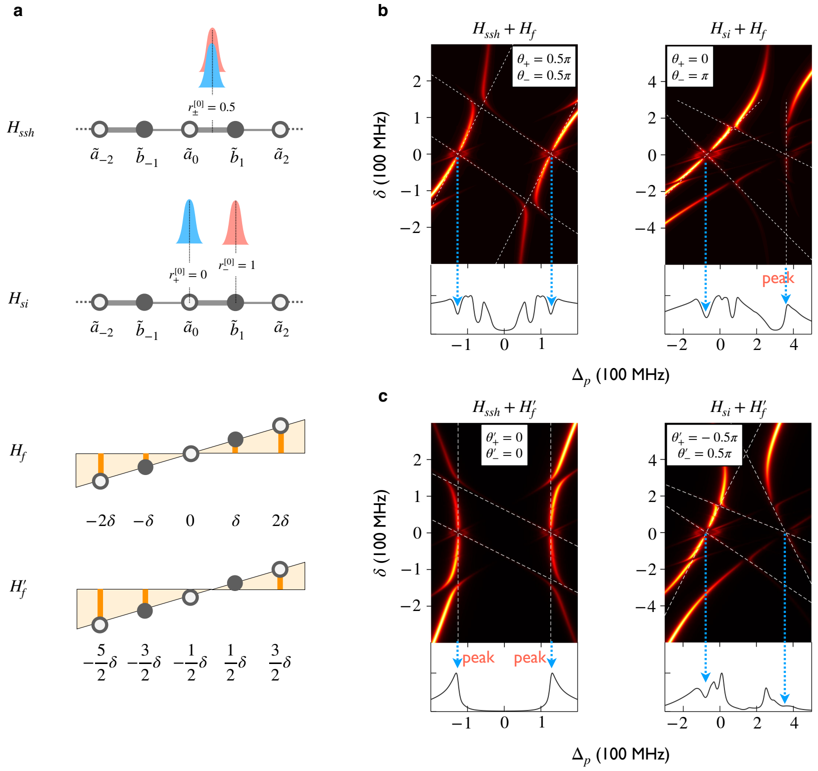

Figure S10. (a) Schematic Wannier centers and linear potentials in the SLs. H_{ssh} (H_{si}) denotes the SSH model (Semenoff insulator) Hamiltonian. H_f and H_f' correspond to potential energies for the same effective force but different zero-energy references. Blue (red) shaded area indicates the position of the upper (lower) band Wannier function in the 0th unit cell. (b) The energy-force diagrams of H_{ssh} and H_{si} with H_f. (c) The energy-force diagrams of H_{ssh} and H_{si} with H_f'.

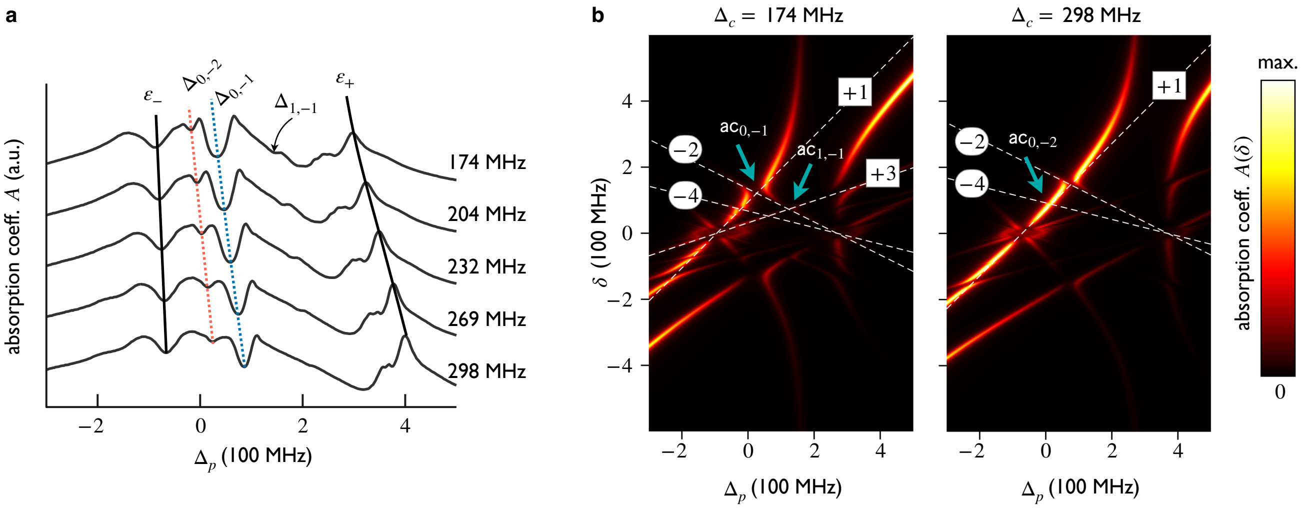

Figure S11. Absorption spectra of the Semenoff insulator superradiance lattices. **(a)** The experimental absorption spectra with $\Omega_{1,2} = 120$ MHz and Δ_c labelled near the data curves. When the coupling is strong enough, higher-order anti-crossings become visible, e.g., the $ac_{1,-1}$ being marked in the data curve with $\Delta_c = 174$ MHz. **(b)** Numerical simulated WSLs and their anti-crossings for $\Delta_c = 174$ MHz and 298 MHz. The coupling between WSLs increases when Δ_c decreases, signatured by the widened dips.

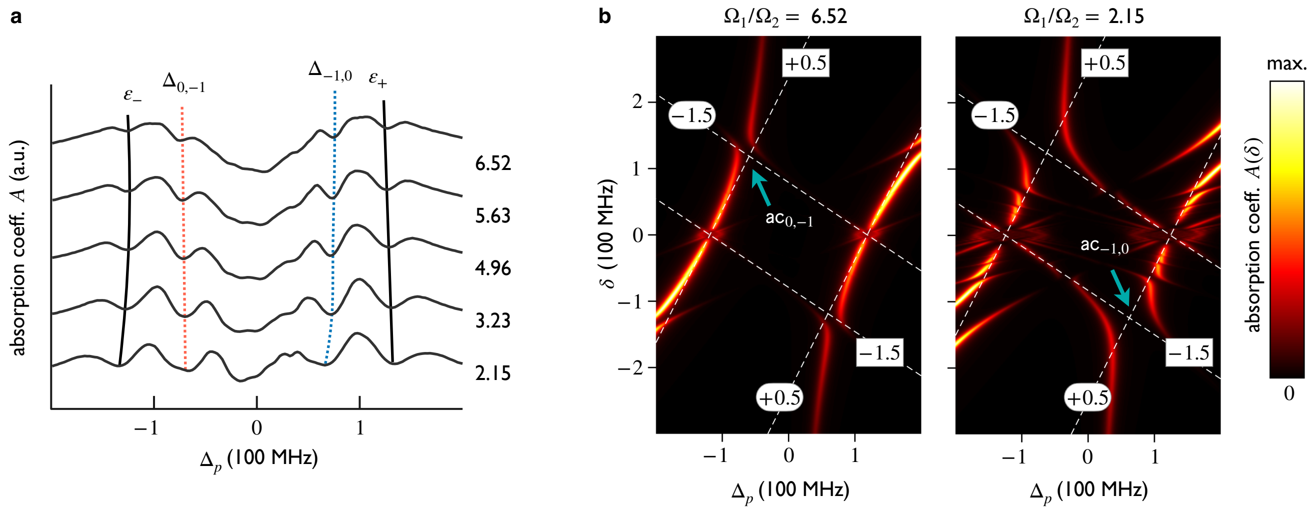

Figure S12. Absorption spectra of the SSH superradiance lattices.. **(a)** The experimental absorption spectra with $\Delta_c = 0$, $\Omega_1 = 118$ MHz and Ω_1/Ω_2 labelled near the data curves. **(b)** Numerical simulated WSLs and their anti-crossings for $\Omega_1/\Omega_2 = 6.52$ and 2.15. The coupling between WSLs increases when Ω_1/Ω_2 decreases.
